# Rapid and sensitive electrochemical sensor of cross-linked polyaniline/oxidized carbon nanomaterials core-shell nanocomposites for determination of 2,4-dichlorophenol

**DOI:** 10.1371/journal.pone.0234815

**Published:** 2020-06-25

**Authors:** Dina F. Katowah, Gharam I. Mohammed, Dyab A. Al-Eryani, Tariq R. Sobahi, Mahmoud A. Hussein

**Affiliations:** 1 Chemistry Department, Faculty of Science, King Abdulaziz University, Jeddah, Saudi Arabia; 2 Department of Chemistry, Faculty of Applied Science, Umm Al-Qura University, Makkah, Saudi Arabia; 3 Department of Chemistry, Faculty of Applied Science, Thamar University, Dhamar, Yemen; 4 Chemistry Department, Polymer Chemistry Laboratory, Faculty of Science, Assiut University, Assiut, Egypt; Texas A&M University at Qatar, QATAR

## Abstract

Nanocomposites (NCs) of crosslinked polyaniline (CPA)-coated oxidized carbon nanomaterials (OXCNMs) were fabricated as a very sensitive and simple electrochemical sensor to be utilized in 2,4-dichlorophenol (2,4-DCPH) detection. CPA/OXCNMs NCs were prepared by chemical copolymerization of polyaniline with triphenylamine and p-phenylenediamine in the presence of OXCNMs. The CPA/GO-OXSWCNTNCs exhibited a higher affinity for the oxidation of chlorophenols compared to the glassy carbon electrode (GCE), CPA/GCE, and other NCs. Cyclic voltammetry was performed to investigate and assess the electrocatalytic oxidation of 2,4-DCPH on the modified GCE. The compound yielded a well-defined voltammetric response in a Britton-Robinson buffer (pH 5) at 0.54 V (vs. silver chloride electrode). Quantitative determination of 2,4-DCPH was performed by differential pulse voltammetry under optimal conditions in the concentration range of 0.05 to 1.2 nmol L^-1^, and a linear calibration graph was obtained. The detection limit (S/N = 3) was found to be 4.2 nmol L^-1^. In addition, the results demonstrated that the CPA/GO-OXSWCNTs/GCE sensor exhibited a strong anti-interference ability, reproducibility, and stability. The prepared CPA/GO-OXSWCNTs/GCE sensor was used to rapidly detect 2,4-DCPH with a high degree of sensitivity in fish farm water with proven levels of satisfactory recoveries.

## 1. Introduction

2,4-dichlorophenol (2,4-DCPH)is a chlorophenol compound that is typically used in herbicides, pharmaceuticals, fungicides, and insect repellents. [1]. However, due to its toxicity, it can be damaging to the environment. In addition, there are several adverse health effects associated with 2,4-DCPH inhalation, such as respiratory infection and liver and kidney damage [2]. Several methods have been utilized to detect 2,4-DCPHincluding spectrofluorimetry [3, 4], electrochemiluminescence [5], spectrophotometry [6], chromatography [7, 8], and electrophoresis [9]. However, these methods are time-consuming and require sample pretreatment. Thus, electrochemical techniques [10–12] have been developed owing to their high sensitivity, simplicity, and inexpensive instrumentation. Consequently, using these techniques, it is possible to create a quick and effective method for detecting 2,4-DCPH in environmental samples.

Carbon nanomaterials (CNMs) have a promising potential for electrochemical applications, such as 2,4-DCPH sensors, as they exhibit high electrical conductivity and large surface areas, while possessing efficient electrocatalytic behavior. In addition, conducting polymer (CP)-based sensors have several advantages including simplicity, ability to process environmental samples, high electrical conductivity, mechanical flexibility, and low cost [13]. Polyaniline (PANI) is a particularly promising CP due to its excellent chemical stability, high electrical conductivity, ease of preparation, low cost, and high yield [14]. Due to its molecular self-assembly, PANI usually forms supramolecular nanofibers with a high surface-to-volume ratio and unique properties, which make it useful in a diverse range of applications. Within the literature, several PANI nanostructures (e.g., nanotubes, nanofibers, and nanospheres) have been prepared using a variety of synthetic techniques [15–18] which has further expanded PANI’s functionality by providing effective designs and improving efficiency. Experimentally, it has been shown that the synergy between individual components improves the features of the NCs, which expands their scope of application [19]. The development of PANI nanostructures and their NCs has introduced a significant potential for the development and optimization of many applications and processes.

Crosslinking can enhance the electrochemical properties of CPs. Consequently, in this study, a chemical in situ polymerization crosslinking method was utilized for the first time in applications to enhance the electrochemical properties of PANI by introducing various types of oxidized carbon nanomaterials (OXCNMs)[graphene oxide (GO), oxidized single-walled carbon nanotubes (OXSWCNTs), oxidized multi-walled carbon nanotubes (OXMWCNTs), (GO-OXSWCNTs), and (GO-OXMWCNTs)] into conjugated crosslinked PANI(CPA). The surface modification of CNMs with carboxylic acid functional groups resulted in noticeable improvements in its characteristics, such as dispersion in the monomer solution. Subsequently, this enhanced the interactions between the matrix and OXCNMs, and increased their conductivity. Therefore, the prepared core-shell NCs combined the advantages of each component which resulted in the development of NCs with electronic conductivity improvements. This led to an electrochemical sensor with considerable selectivity and sensitivity for 2,4-DCPH (kindly refer to the graphical abstract associated with this submission).

## 2. Materials and methods

### 2.1. Materials

Aniline (ANI) was procured from Shanghai Chemical Reagent Co.(China), and ammonium persulfate (APS) was procured from Across Organics (Belgium).p-phenylenediamine (PPDA) was purchased from PDH and used without further purification(UK). Triphenylamine (TPA) was procured from Janssen Chimica (Belgium). 2,4-DCPH was obtained from Aladdin Chemistry Co. (Shanghai, China). Phenol (BDH) was purchased from Analar Limited (UK). 2-chlorophenol (2-CP), o-phenylenediamine (o-PDA), and 4-chlorophenol (4-CP) were procured from BDH laboratory reagents(UK). Ethanol, dimethylformamide (DMF), sulfuric acid (H_2_SO_4_), and nitric acid (HNO_3_) were procured from Sigma-Aldrich(USA). Graphene, SWCNTs, and MWCNTs were procured from the XFNANOA dvanced Materials Supplier Inc. (China). Additionally, analytical-grade chemicals were used in the experiments as received. Ultrapure, distilled, and deionized water (DI water)were used throughout the experiments. A series of Britton-Robinson (B-R) buffers (pH 2–11) were synthesized according to a previously reported method [20]. Glassware, bottles, and other instruments were pre-cleaned with chromic acid (H_2_CrO_4_), washed with B-R buffer(10%, v/v) and acetone, and rinsed with distilled water.

### 2.2. Instrumentation

The degree of crystallinity of the samples was characterized by x-ray diffraction (XRD, D8, Bruker). The morphologies of the prepared materials were evaluated by scanning electron microscopy (SEM, Quanta 250 FEG, Thermo Fisher) and high-resolution transmission electron microscopy (HR-TEM, JEM-2100, JEOL). Thermogravimetric analysis (TGA) and differential thermal gravimetry (DTG) tests were carried out to assess the thermal stability of the NC materials. It was also necessary to standardize their degradation temperature. Therefore, analysis was conducted on a TGA-50 system at a heating rate of 10 °Cmin^-1^ in air. The functional group information of the samples was obtained by Fourier-transform infrared spectroscopy (FTIR,JASCO). Raman spectroscopy was recorded on a Lab RAM-HR(Evolution Horiba Co.). Electrochemical measurements were carried out using a Metrohm 757 VA trace analyzer and a 747 VA stand (Switzerland). A three-compartment borosilicate (Metrohm) voltammetric electrochemical cell (100 mL) configuration incorporated a modified glassy carbon electrode (GCE, Φ = 3 mm) as the working electrode, a silver chloride electrode (AgCl/Ag) as the reference electrode, and a platinum wire as the counter electrode. Ultrapure water (Milli-Q Plus, Millipore) was used for the preparation of all solutions. Digital micropipettes [0.5–10 μL (Joan-Lab), 10–100 μL and 100–1000 μL (Plus-Sed)] were used. Solution pH values were measured using a pH meter (inoLap pH/ion level 2).

### 2.3. Synthesis of OXCNMs

#### 2.3.1. Synthesis of GO

GO was synthesized as described by the modified Hummers’ method [21].

#### 2.3.2. Synthesis of OXSWCNTs and OXMWCNTs

OXSWCNTs were carefully prepared in accordance with a method outlined within the literature [22]. SWCNTs (100 mg) were dispersed in a mixture (1:3, v/v) of HNO_3_)70%(and H_2_SO_4_(96%)and ultrasonicated for 4 h. Next, the suspension was refluxed for 2 h in an oil bath at 80°C under stirring. The resulting OXSWCNTs were diluted with DI water until the washing solution gave a pH > 5, then they were filtered and dried in an oven at 60°C. OXMWCNTs were prepared by the same process using MWCNTs.

#### 2.3.3. Synthesis of GO-OXSWCNTs and GO-OXMWCNTs

To prepare GO-OXSWCNTs, OXSWCNT powder was added to 25 mL of GO dispersion in DI water ata mass ratio of 1:1 and sonicated for 2 h. The obtained dispersion was filtered and dried at 100°C for 24 h. GO-OXMWCNTs were prepared by the same process using OXMWCNTs.

### 2.4. Synthesis of CPA and CPA/OXCNM NCs

The chemical copolymerization of CPA with various OXCNMs was carried out using the following method. First,5%OXCNMs were added to 120 mL of 0.5 M H_2_SO_4_ within a three-neck flask (250 mL) and the mixture was ultrasonicated for 2 hat room temperature. Then, 1.8626 mL of doubly distilled ANI, 0.043256 g of PPDA, and 0.049064 g of TPA were added to the flask and the mixture was ultrasonicated for approximately 30 min. The solution was then continuously stirred and cooled to 0–4°C in an ice bath. Then, 5 g of a pre-cooled APS solution (dissolved in 40 mL of 0.5 M H_2_SO_4_) was added dropwise to the solution over approximately 30 min under a nitrogen atmosphere with constant stirring at 0–4°C. Polymerization was continued for 24 h under a nitrogen atmosphere while the solution was stirred constantly at 0–4°C. The obtained precipitate was then collected by ultracentrifugation and washed using DI water repeatedly until the resulting filtrate became colorless. Finally, the fine black powder was dried at 60°C for 24 h. This preparation procedure was repeated five times at fixed loadings of various OXCNMs (GO, OXSWCNTs, OXMWCNTs, GO-OXSWCNTs, and GO-OXMWCNTs). For comparison, pure CPA was synthesized by the same method without OXCNM [18]. The abbreviations for the prepared CPANCs are defined as CPA/GO, CPA/OXSWCNTs, CPA/OXMWCNTs, CPA/GO-OXSWCNTs, and CPA/GO-OXMWCNTs.

### 2.5. Fabrication of the working electrode

To fabricate the working electrode, the GCE was first polished to a mirror finish with a 0.05 μm alumina slurry in ultrapure water using a smooth polishing material and applying circular movements for a few minutes. The surface was then washed with ultrapure water and sonicated in HNO_3_ (10%, v/v), ethanol, and ultrapure water for 1, 2, and 3 min, respectively. The formation of a GCE modified with NCs was achieved using drop-coating, where 10 mg of the NCs were dispersed in 5 mL of DMF [23]. Then, high-frequency ultrasonication was used to prepare homogeneous NC suspensions. A total of 6 μL of NC suspension was dropped onto the GCE and on the surface of bare GCE, followed by vacuum drying at 50°C for 15 min [24].

### 2.6. Electrochemical measurements

Nitric acid (10%,v/v) and ethanol were utilized to clean the electrochemical cell which was later washed with ultrapure water. All electrochemical experiments were performed at room temperature. Voltammetric measurements were performed in a 10mL electrochemical cell containing 2,4-DCPH (1.0 × 10^−4^ mol L^−1^) as the target analyte and B-R buffer (pH 5) as the supporting electrolyte. Cyclic voltammograms between -0.50 and 1.2 V were measured at a scan rate of 50 mV s^-1^. The voltammetric cell and test solution were purged with nitrogen and stirred for 15 min. After 10 s of equilibration, the background voltammogram of the supporting electrolyte was recorded by applying a potential scan from -0.5 to 1.2 V vs. Ag/AgCl at 0.05s modulation time, 0.5s interval time, 0.00195V step potential, 0.07995V modulation potential, and -1.0V standby potential. Under the same experimental conditions, the effect of the sweep rate (ν = 0.02.0.16 V s^−1^) on the cyclic voltammograms of 2,4-DCPH(1.0 × 10^−4^ mol L^−1^) at pH 5 was determined. The 3-electrode system was immersed in a blank solution after each measurement. Then, the cyclic voltammetry (CV) scan was repeated 20 times to renew the electrode.

### 2.7. Water sample preparation and analysis

Water samples collected from a fish farm basin (Faculty of Marine Sciences, King Abdulaziz University, Jeddah, Saudi Arabia) were stored at 5°C. Before analysis, the water samples were filtered through a 0.50μmmembrane filter and transferred to volumetric flasks filled with ultrapure water. A required quantity of the water samples was diluted with a B-R (pH 5.0) buffer up to 10 mL for electrochemical measurements. The differential pulse voltammetry(DPV)signals were recorded under optimum conditions after deaeration with nitrogen.

### 2.8. Ethics statement

In this research work, no permits were required to perform. We are a university staff and doing our research work in the university laboratories. Therefore we have no permission to do any regular experiments. In addition our research has no any medical treatments or no deal with animals.

## 3. Results and discussion

### 3.1. Characterization

**Fig 1:**
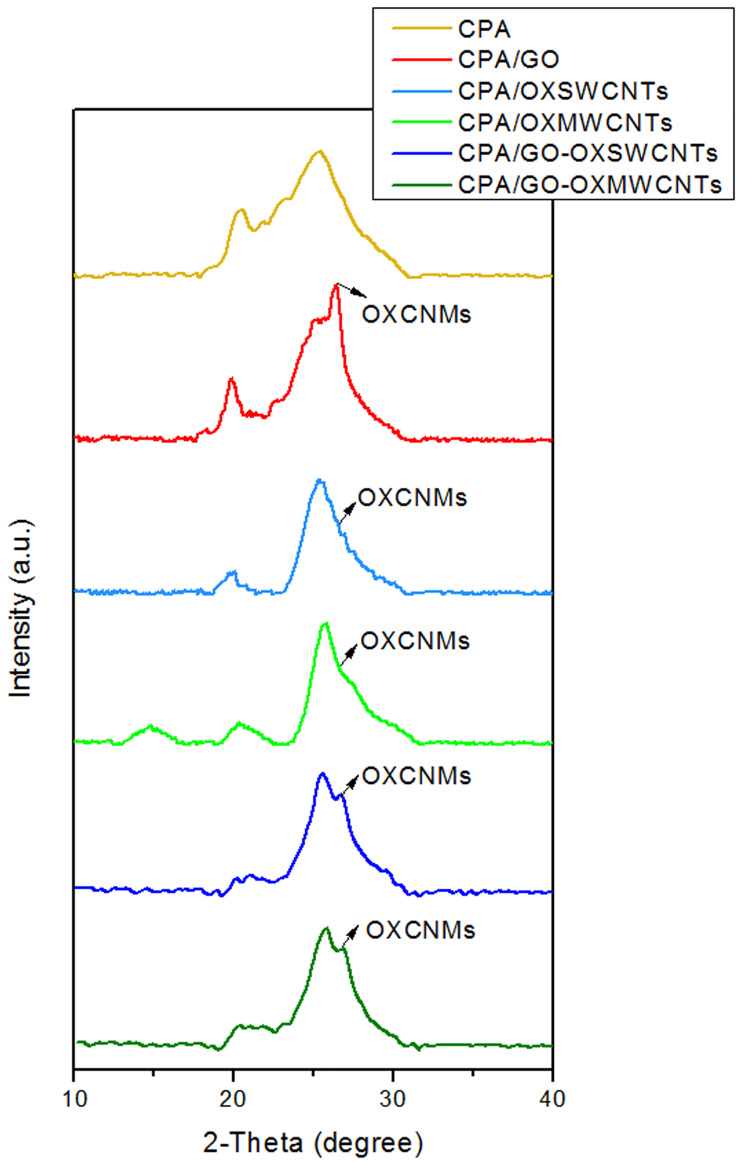
XRD patterns of CPA and CPA/OXCNMsNCs.

Fig 1 shows the XRD diffraction patterns of CPA and as-prepared CPA-OXCNM NCs. The CPA exhibited two main reflections located at 2θ = 20.4 and 25.4°, which is in agreement with the reflection angles obtained in previous studies of CPA [25]. Typically, the patterns of all CPA-OXCNM NCs exhibit the same behavior and they have similar diffraction peaks as those of pure CPA. However, the peaks appear sharper and more intense, suggesting that NCs are more crystalline and the polymer chains in the NCs have an ordered structure. The presence of a crystalline peak in all NCs at approximately 26° with a higher intensity is due to the overlapping peaks of CPA and graphitic materials in the OXCNMs. Consequently, this enhances long-range conjugation and improves π-π inter chain stacking [26, 27]. In addition, the appearance of these peaks indicates the formation of the core-shell structure of the CPA-OXCNMs, as evidenced by the SEM image.

**Fig 2:**
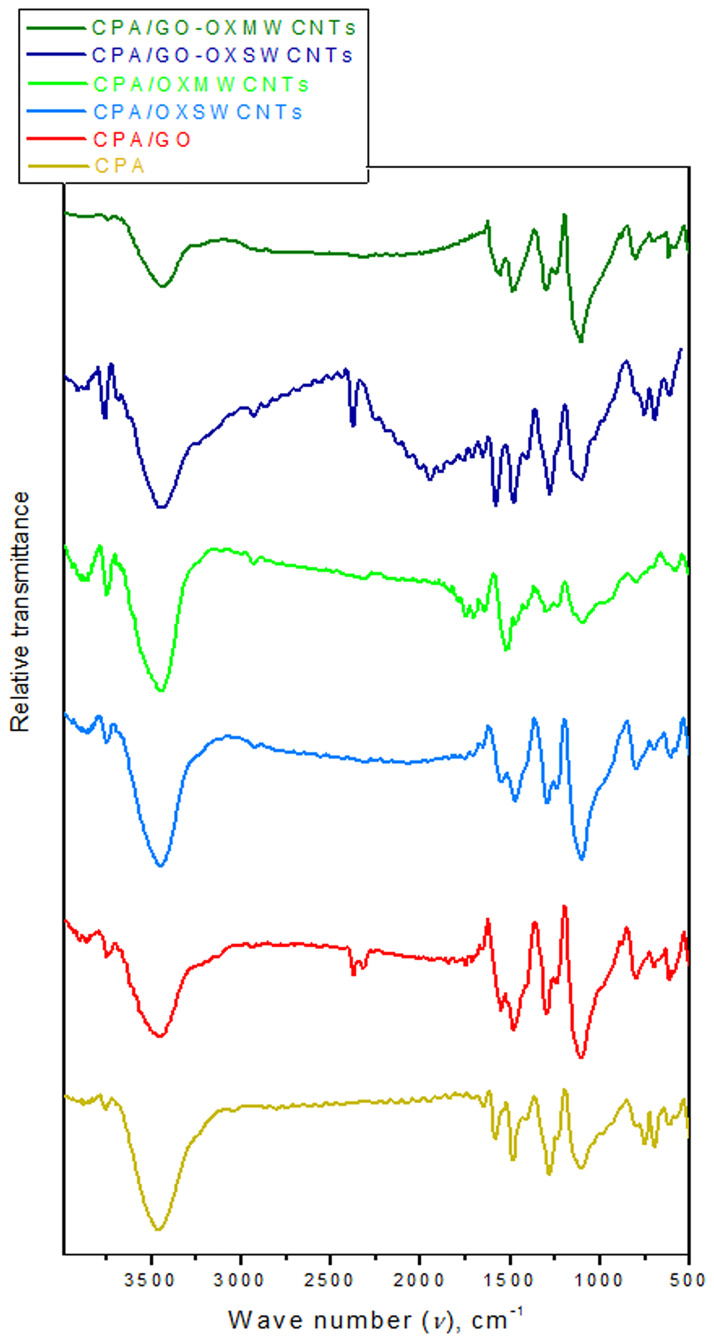
FTIR of CPA and CPA/OXCNMsNCs.

Fig 2 shows the FTIR spectra of CPA, which is in agreement with previous results from the literature [25]. The peak at approximately 1580 cm^-1^ is due to C = C stretching vibrations of the quinonoid rings, while the peak at approximately1483 cm^-1^ is ascribed to C = C stretching vibrations of the benzenoid units. The stretching mode of C–N, which belongs to aromatic amine, is observed at 1306 and 1240 cm^-1^. The strongest peak at approximately 1111 cm^-1^ is a characteristic peak of the doped structure; this strong peak occurs due to the high doping level, which increases conductivity [28, 29]. The peak attributed to the out-of-plane C–H bending vibration in the 1,4-disubstituted rings is located at 800 cm^-1^ [30]. The peak centered at 1645 cm^-1^ is related to the stretching vibration of aromatic rings; it is prominent because of the increase in the content of benzenoid as a result of the copolymerization of TPA as well as PPDA [31]. The stretching mode of N–H in CPA is located at 3450 cm^-1^. The spectra of the NCs show some differences when compared to those of CPA. In the spectra of the NCs, the intensities of the peaks decreased, indicating an interaction between CPA chains and OXCNM surfaces [32]. The peak at 1111 cm^-1^, which is attributed to the doped CPA, is a measure of the degree of delocalization of electrons [33]. Thus, it is the characteristic peak of the CPA electrical conductivity. The intensity of this peak increased with the introduction of the OXCNMs, and the peak was slightly shifted. This finding supports the existence of strong interactions between the π-bonded structure of the OXCNMs and the conjugated structure of CPA [34, 35], and the shift is caused by the electrostatic interactions between CPA and OXCNTs. This shift is similar to that observed in the Raman spectra and the XRD pattern of the NCs (Fig 1), where the peak at 26° in the NCs was tapered and more intense compared to that of CPA. The intensity of this peak was the highest for the CPA/GO-OXSWCNTs compared with those of other NCs, which confirms its high conductivity. This result is in good agreement with the measured increased electrical conductivity.

**Fig 3:**
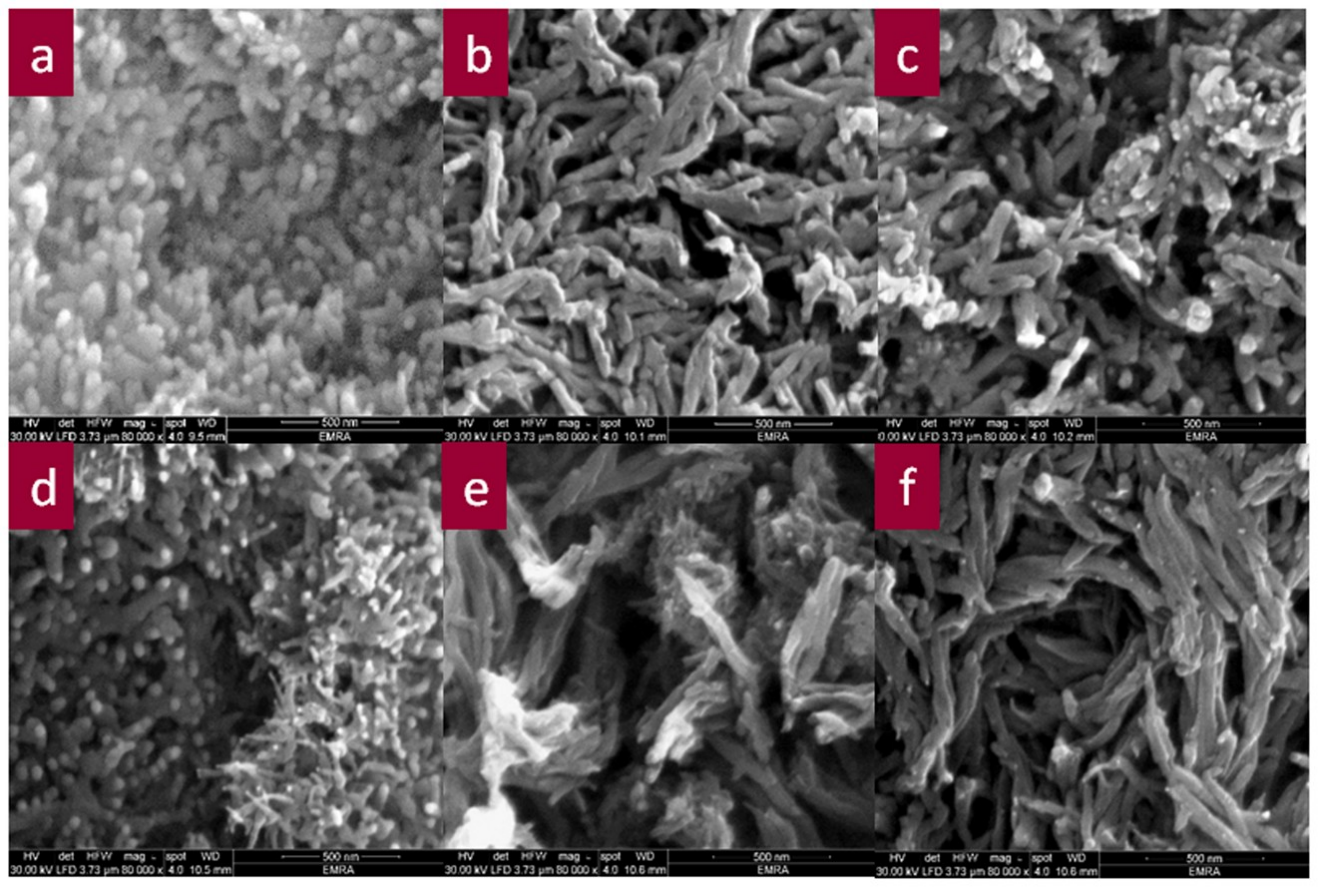
SEM images of CPA (a), CPA/GO (b), CPA/OXSWCNTs (c), CPA/OXMWCNTs (d), CPA/GO-OXSWCNTs (e) and CPA/GO-OXMWCNTs (f).

Surface morphology is a crucial factor that affects the electrochemical performance of electrode materials in sensors. Fig 3 shows SEM images of pure CPA and CPA/OXCNM NCs with various OXCNM contents. The CPA sample exhibited a nanorod-like morphology, as shown in Fig 3a. For CPA/OXCNM NCs, during in situ polymerization, OXCNMs served as templates and CPA nanorods evenly grew on their surfaces via electrostatic interactions, π-π electron interactions, and hydrogen bonding between OXCNMs and CPA chains as well as between CPA chains. These interactions produced well-aligned nanorod core-shell structures, as shown in Fig 3b–3f with large surface areas, which may enhance electrical conductivity when these structures are used as electrode materials [36, 37]. For CPA/GO-OXSWCNTs and CPA/GO-OXMWCNTs, the increase in their conductivity is attributed to the mixture of GO-OXSWCNTS and GO-OXMWCNTs, respectively. The presence of these materials yields highly interconnected networks in both NCs. This, in turn, offers fast electron charge transportation paths. Thus, the ionic mobility and diffusion pathways are improved, which increases the electrical conductivity.

**Fig 4:**
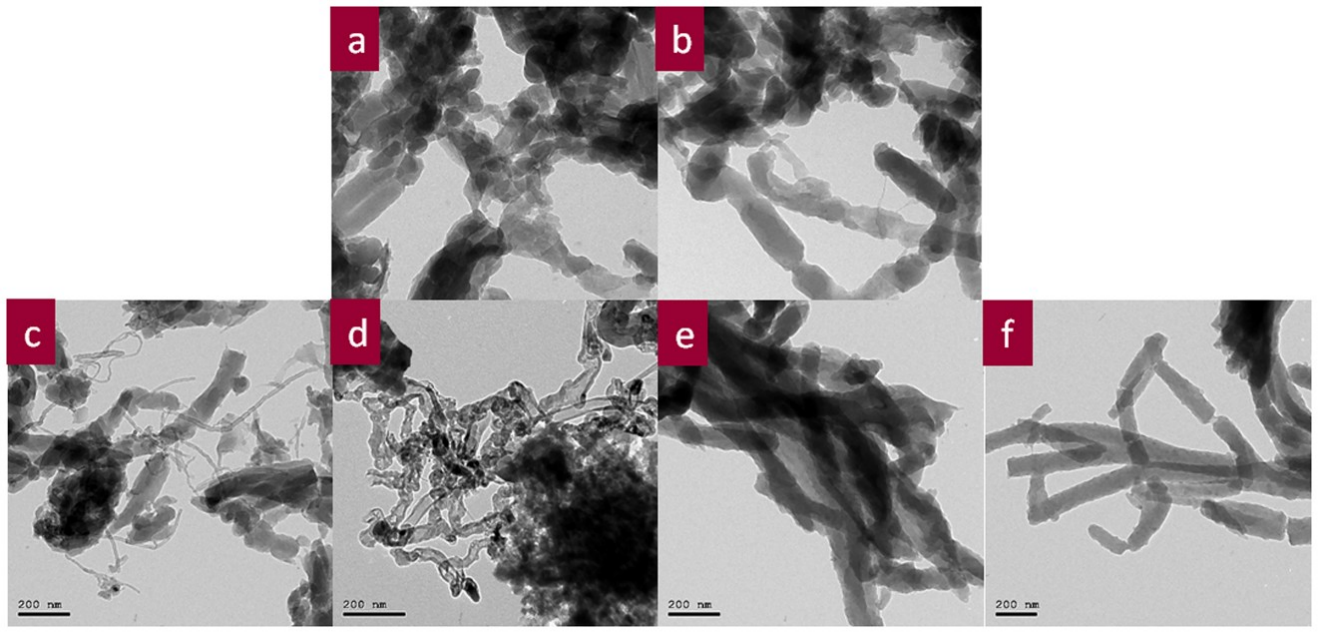
TEM images of CPA (a), CPA/GO (b), CPA/OXSWCNTs (c), CPA/OXMWCNTs (d), CPA/GO-OXSWCNTs (e) and CPA/GO-OXMWCNTs (f).

The HR-TEM images of CPA and NCs show regular nanorod-like morphology [Fig 4a–4f and 4c–4f] with both types of CNTs. It is clear that the thickness of the CNTs increased due to the formation of the core-shell nanostructure of CPA-coated CNTs. No GO was observed in the CPA/GO, CPA/GO-SWCNTs, and CPA/GO-MWCNT NCs, as shown in Fig 4b, 4e and 4f, respectively. It can be concluded that the CPA covered the GO completely. GO may serve as a substrate to support CPA-coated OXCNTs during polymerization in both CPA/GO-OXSWCNTs and CPA/GO-OXSWCNT NCs. This yields an increased surface area, and therefore increased electrical conductivity of the CPA/GO-OXSWCNTs and CPA/GO-OXSWCNT NCs compared with that of other NCs (confirmed from the thickness of NCs by SEM and HR-TEM).

**Fig 5:**
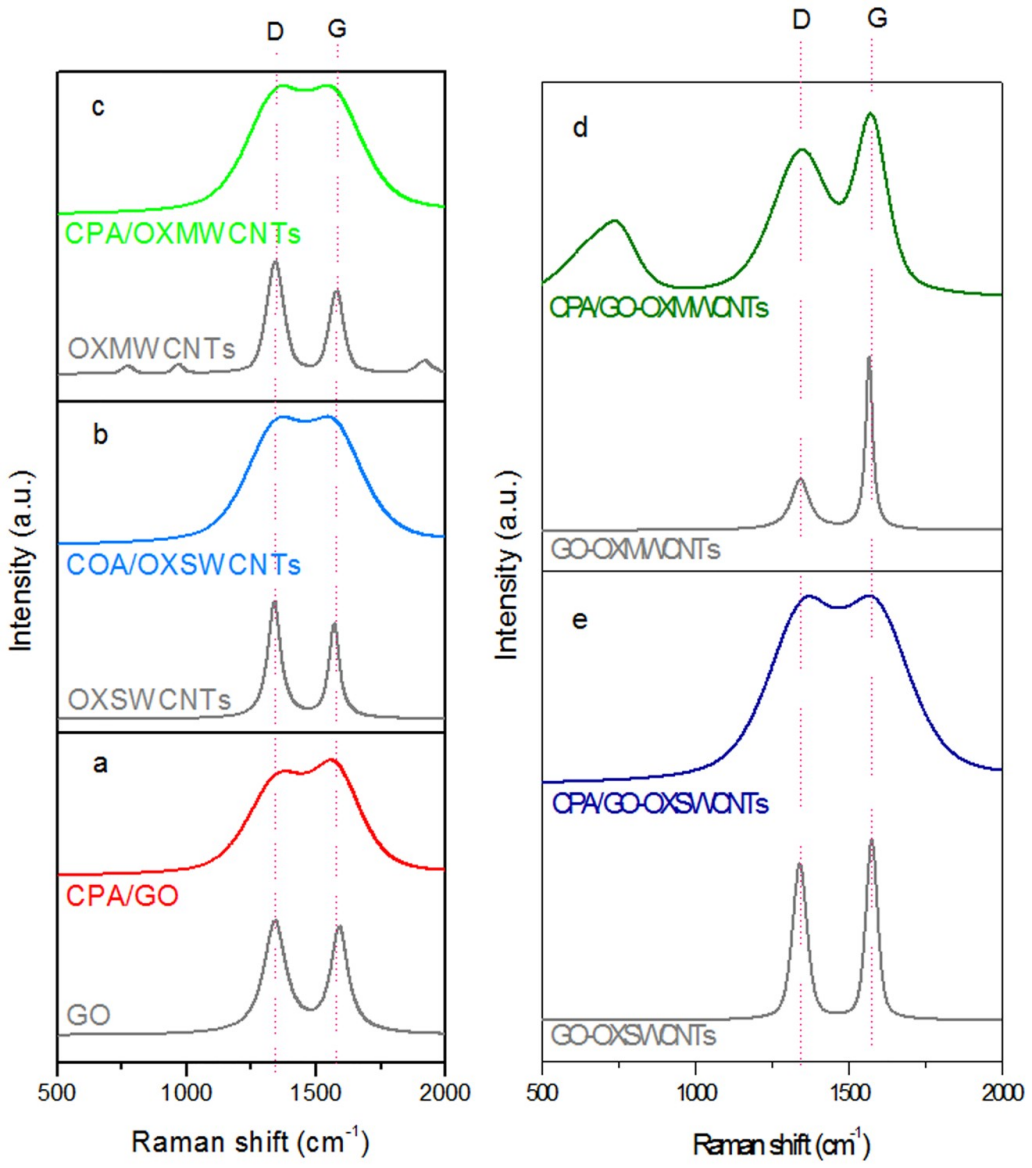
(a and b) RAMAN of OXCNTs and CPA/OXCNMsNCs.

Raman spectroscopy was used to confirm the existence of OXCNMs side-by-side with CPA in the NCs. Fig 5a and 5b show the Raman spectra of OXCNMs and CPA/OXCNM NCs, respectively. The characteristic peaks of D and G of the OXCNMs appear in the Raman spectra of the NCs. The G band represents the in-plane stretching E_2g_ mode, whereas the D band is disorder-induced, for example, by sp^3^-hybridized carbon atoms [38]. Fig 5a shows the Raman spectrum of the GO nanoparticles (NPs). The characteristic features in the Raman spectrum of the GO NPs are the D and G bands, which are centered at approximately 1342and 1592 cm^-1^, respectively. The characteristic peaks of D and G for the GO NPs are present in the Raman spectrum of the CPA/GO NCs. The D and G bands of the CPA/GO NCs are slightly shifted compared with those of the GO NPs and appear at 1380 and 1554 cm^-1^, respectively. Fig 5b shows the spectrum of pure OXSWCNTs, which has a strong G band at 1571 cm^−1^ and a D band at 1340 cm^−1^. However, the G band of OXSWCNT at 1571 cm^−1^ was shifted to lower values (i.e., to 1543 cm^−1^ for CPA/OXSWCNTs), whereas the D band shifted from 1340 to 1375 cm^-1^.

The Raman spectrum of the OXMWCNTs is shown in Fig 5c, where the D and G bands were observed at 1342 and 1578 cm^-1^, respectively. However, the D and G bands for the OXMWCNTs were shifted from 1342 to 1373 cm^-1^ and from 1578 to 1543 cm^−1^, respectively, for the CPA/OXMWCNTs. The spectrum for the GO-OXSWCNTs [Fig 5d] demonstrates that the D and G bands are centered at 1343 and 1579 cm^-1^, respectively. The characteristic G peak of the GO-OXSWCNTs at 1579 cm^-1^ is present in the Raman spectrum of the CPA/GO-OXSWCNT NCs. The G band of the CPA/GO-OXSWCNT NCs is slightly shifted compared with the GO-OXSWCNTs and appears at 1571 cm^-1^, whereas the D band at 1343 cm^-1^ is shifted to 1378 cm^-1^. Similarly, the GO-OXMWCNTs spectrum [Fig 5e] shows the D and G bands at 1343 and 1568 cm^-1^, respectively. The characteristic G peaks of GO-OXMWCNTs at 1568 cm^-1^ are present in the Raman spectrum of the CPA/GO-OXMWCNT NCs, but the G band is slightly shifted and appears at 1571 cm^-1^. The D band is also shifted from 1343 to 1349 cm^-1^. The values of the G and D bands of the NCs are summarized in Table 1.

**Table 1:** The values of G, D band and ID/IG ratio of OXCNMs and CPA/OXCNMsNCs.

	OXCNMs			NCS		
NCS	G	D	ID/IG	G	D	ID/IG
CPA/GO	1592	1342	0.842	1554	1380	0.888
CPA/OXSWCNTs	1571	1340	0.852	1543	1375	0.891
CPA/OXMWCNTs	1578	1342	0.850	1543	1373	0.889
CPA/GO-OXSWCNTs	1579	1343	0.850	1571	1378	0.877
CPA/GO-OXMWCNTs	1568	1343	0.856	1571	1349	0.858

The shifting of the G band in the Raman spectra for all of the NCs indicates that a strong interaction characterized by charge transfer occurs between the OXCNMs and CPA. The broadening of the G band of the CPA/OXCNM NCs may be due to the coating of amorphous CPA which forms on the OXCNMs during synthesis [39, 40]. This is further supported by the thicker coating of CPA on the OXCNMs, as confirmed by SEM. The intensity ratio (I_D_/I_G_) of the D and G bands is an important parameter for measuring the degree of graphitization in the material. The difference in the given intensity ratio of the pristine OXCNMs and NCs is a good indicator of the relative degree of defects in the OXCNMs. If both bands have similar intensities, a high level of structural defects may be present in the OXCNM lattice [41]. As shown in Table 1, the intensity ratios of all the NCs were greater than the OXCNMs, which demonstrates that no defects were created in the OXCNM lattice.

**Fig 6:**
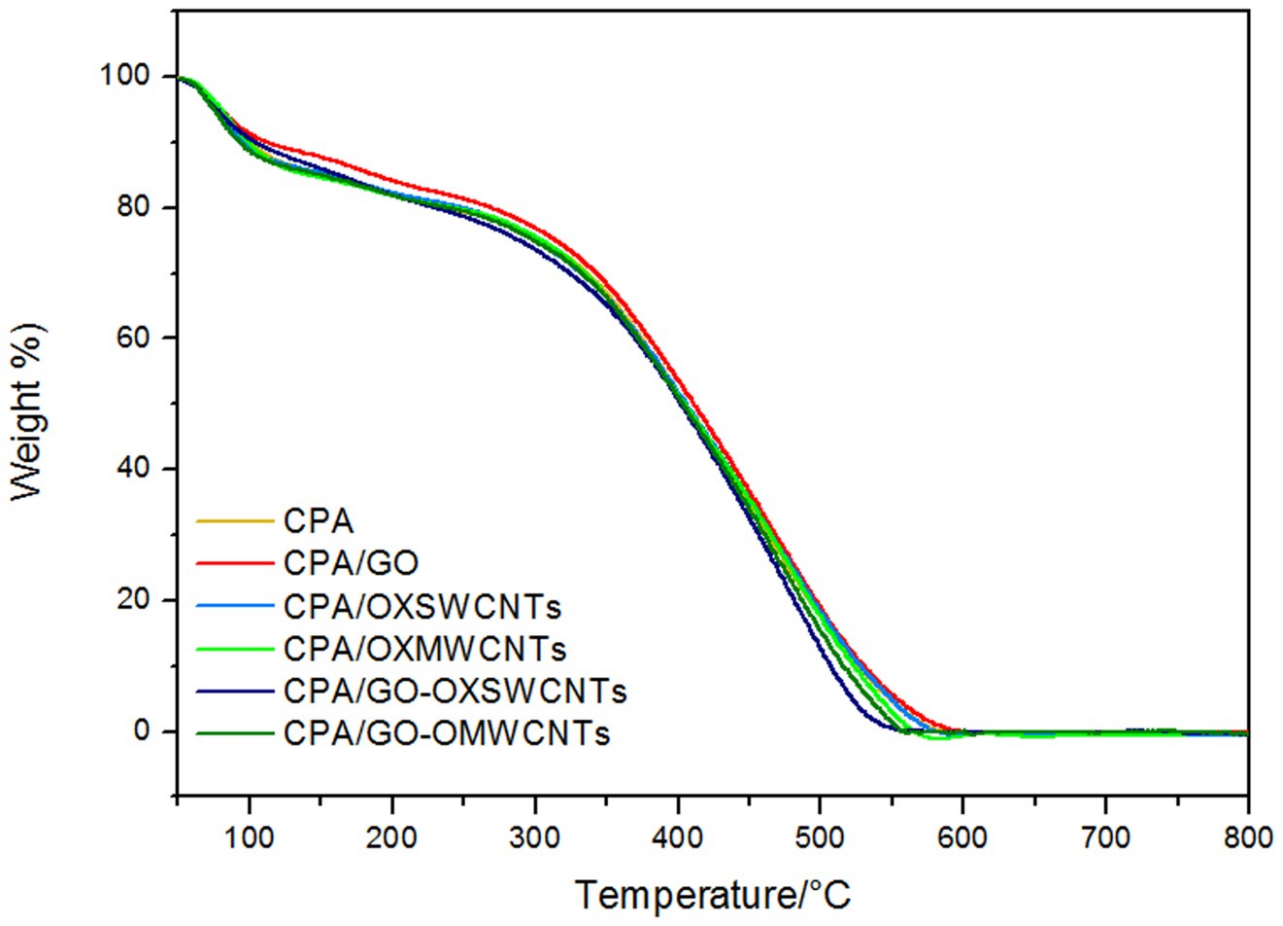
TGA curves of CPA and CPA/OXCNMsNCs.

The CPA and CPA/OXCNM NC samples were characterized by TGA and DTG to examine their thermal stability and weight loss. Fig 6 and S1 Fig show the TGA curves for all samples as a function of temperature together with the DTG curves. The weight loss of CPA observed in the TGA curves occurred in three steps, which agreed with the literature [25]. The first step of weight loss (14.1 wt.%) started at 50–140°C and was caused by the evaporation of moisture. The second step of weight loss (5.7 wt.%) occurred at 150–240°C and was attributed to the removal of doping anions. In addition, it may also be attributed to the rapid degradation of poly(p-phenylenediamine) [42] through the homopolymerization of PPDA during copolymerization crosslinking. The weight loss in the third step occurred at 240–640°C and was mainly attributed to the decomposition of the polymer chains. Complete decomposition of CPA was observed at 591°C. The CPA/OXCNM NCs show a behavior similar to that of CPA, which suggests that these samples exhibit a similar degradation process, as shown in Fig 6.

In Table 2, the notationsT_10_, T_25_, and T_50_ representthe decomposition temperatures which correspond to 10, 25, and 50% weight loss, respectively. The T_10_, T_25_, and T_50_ values for the CPA/GO NCs were 111, 314, and 414°C, respectively. This signifies that, compared with the other samples, the CPA/GO NCs had the highest thermal stability and residual mass retention. The final composite degradation temperature (CDT_*final*_)is defined as the temperature at which decomposition finishes [43]. The values of CDT_final_ obtained from the TGA curves are displayed in Table 2, and exhibit that the CDT_*final*_ of the CPA/GO NCs is higher than that of the other samples. The maximum polymer degradation temperature (PDT_*max*_) [44] values were determined for all samples, and the values are shown in Table 2. The results in Table 2 also demonstrate that the interactions between CPA and the mixture of GO-OXSWCNTS have the highest value compared to the other NCs. This may potentially be due to the presence of OXSWCNTs in the GO-OXSWCNTS mixture.

**Table 2:** Thermal behaviour of CPA and CPA/OXCNMsNCs.

Sample	Temperature (°C) for various percentage decompositions ^a^			PDT_*max*_^b^ (°C)	CDT_*final*_ ^a^ (°C)
	T10	T25	T50		
CPA	98	302	405	461	591
CPA/GO	111	314	414	467	597
CPA/OXSWCNTs	95	298	407	469	580
CPA/OXMWCNTs	99	304	404	473	565
CPA/GO-OXSWCNTs	102	288	401	482	558
CPA/GO-OXMWCNTs	92	298	403	474	559

^a^ The values were determined by TGA at a heating rate of 10 °C min^−1^

^b^ Determined from the DTG curves.

**Fig 7:**
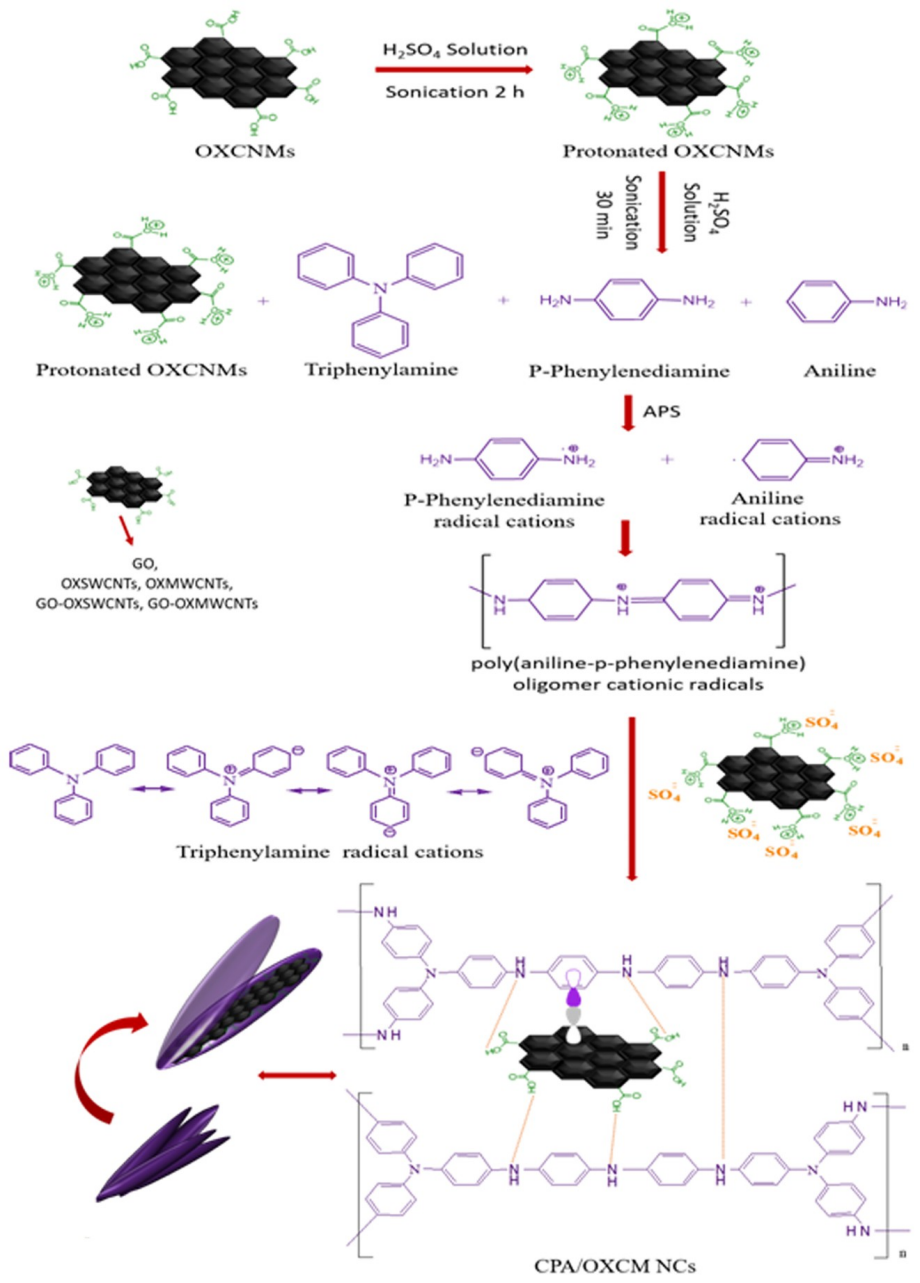
Illustration of the fabrication procedure of core-shell PAC/OXCNMsNCs.

Given the aforementioned analysis, a scheme for the fabrication of novel CPA/OXCNM NCs is presented to highlight the synthesis method of the NCs (Fig 7). Facile chemical oxidative polymerization was applied to prepare the CPA/OXCNM NCs in the presence of OXCNMs in an acidic medium; TPA was used as a crosslinker in the polymerization of ANI with PPDA. In an acidic environment, the groups of -COOH on the surface of the OXCNMs tend to become protonated, indicating a potential gain in H^+^ from the acidic medium [45]. Thus, several SO_4_^2−^ions compensate for the positive charges on the surface of the OXCNMs by adsorption. In addition, extra adsorption of SO_4_^2−^ on the surface of the OXCNMs acts as a charge compensator for positively charged CPA chains during the formation of the CPA/OXCNM NCs in this charge compensation system. ANI, PPDA, and TPA were oxidized by APS into cationic radicals under acidic environments. The polymerization of the cationic radicals of ANI and PPDA create linear poly(ANI-PPDA) oligomers with two amino groups in the terminal. After oxidation, the cationic radicals of the linear poly(ANI-PPDA) oligomer continue to react with cationic radicals of TPA to form a CPA network. The polymerization occurs equally in the three para-positions because of the identical reaction activity of the three para-positions of N in TPA. Electrostatic interactions occur between cationic radicals and anions absorbed on the surface of the OXCNMs. Furthermore, we propose that two types of hydrogen bonding occur: bonding between CPA chains and oxygen atoms on the surface of OXCNMs and hydrogen bonding between CPA chains in the CPA/OXCNM NCs. Additionally, π–π bonding between the aromatic rings of CPA and the π bonds of OXCNMs also occurs. These interactions confirm that OXCNMs are inserted into CPA chains and produce core-shell structures. These interactions generate conducting bridges that facilitate higher charge transfer between the quinoid ring of CPA and the OXCNMs, which enhances the electrical conductivity of the NCs [25, 46–50].

### 3.2. Electrochemical characterization of modified electrodes

**Fig 8:**
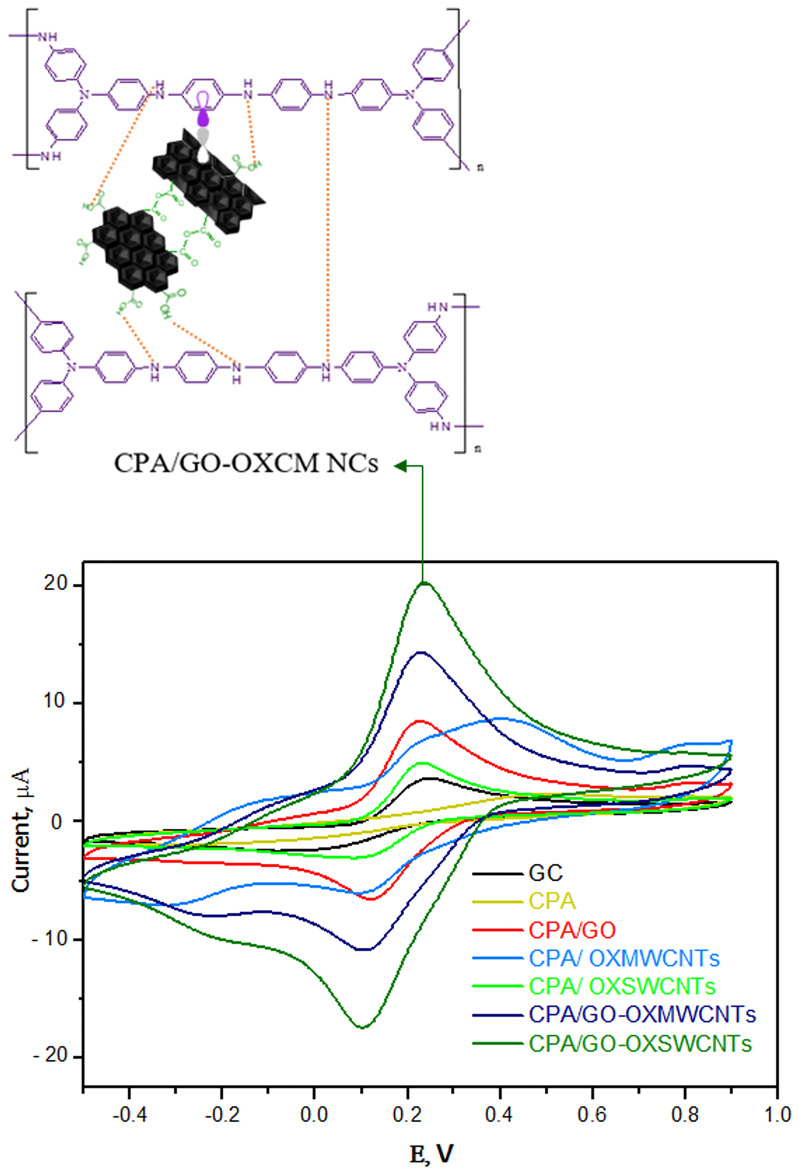
The CV curves for bare GC electrode, CPA/GC and CPA/OXCNMs/GC electrode in 0.1 M KCl solution containing 5.0 mM [Fe(CN)6]^3-/4-^ with the scan rate of 50 mVs^-1^.

CV used ferricyanide as a redox probe to measure the surface status as well as the barrier of the modified electrodes, and the corresponding cyclic voltammograms are shown in Fig 8. A clear well-defined reversible redox peak was observed in the CV curve of the bare GCE because of the [Fe(CN)_6_]^3−/4−^ electron transfer process. When the GCE was modified with CPA, a sharp decrease in peak currents was observed. This occurred because CPA has poor conductivity compared to other samples, which hinders electron transfer. CPA/GO, CPA/OXMWCNT, and CPA/OXSWCNT electrodes reported a small increase in peak currents. For CPA/GO-OXMWCNTs, the redox peak currents of [Fe(CN)_6_]^3−/4−^ were considerably improved. This is because the CPA/GO-OXMWCNTs have a large specific surface area, and subsequently a high electrical conductivity, which increases the concentration of [Fe(CN)_6_]^3−/4−^ on the electrode surface and promotes a high electron transfer rate. For CPA/GO-OXSWCNTs, the currents were further increased, which indicates a synergistic effect between the CPA and GO-OXSWCNTs. This improves the electron transfer of [Fe(CN)_6_]^3−/4−^ on the surface of the electrode.

### 3.3. Electrochemical performance of the modified electrodes in detecting2,4-DCPH

**Fig 9:**
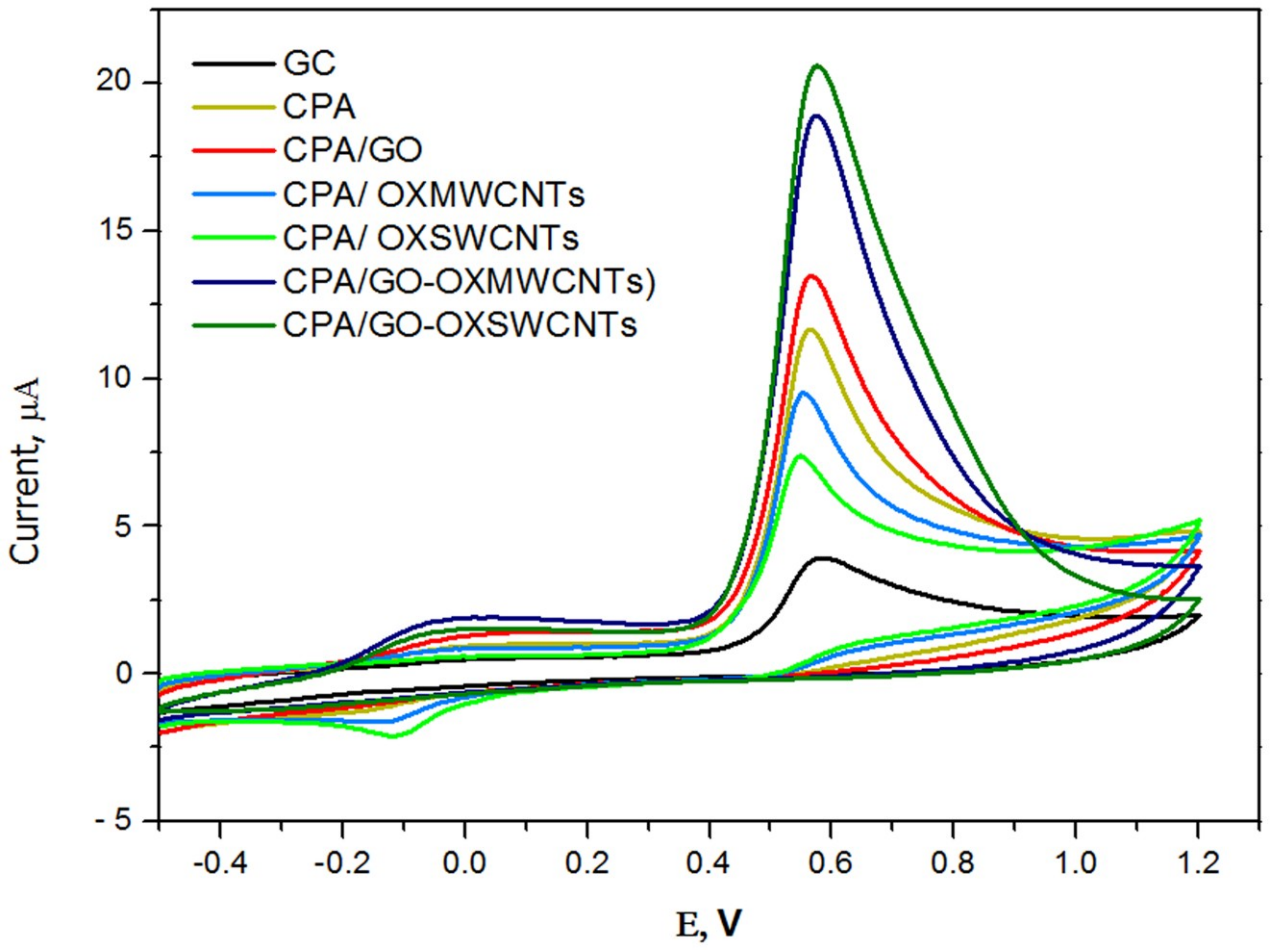
CVs of 0.1 mM 2,4-DCP at GCE, CPA/GC and CPA/OXCNMs/GC in B-R (pH = 5.0) with the scan rate of 50 mVs^-1^.

The sensing performance of the prepared modified electrodes for 2,4-DCPH was evaluated by CV. Fig 9 displays the voltammetric responses of 2,4-DCPH (1 × 10^−4^ mol L^-1^) in B-R buffer (pH 5.0) at different electrodes. A weak oxidation peak at 0.58 V with a small peak current of 3.92 μA was observed for the bare GCE. The CPA/GCE and CPA/GO/GCE had slightly higher oxidation currents (11.7 and 13.5 μA, respectively) and a marginally lower potential (0.56 V) compared to the bare GCE. The CPA/OXMWCNTs/GCE and CPA/OXSWCNTs/GCE exhibited the lowest oxidation peak currents (9.5 and 7.4 μA, respectively) at 0.55 V. The CPA/GO-OXMWCNTs and CPA/GO-OXSWCNTs, where the films accelerate 2,4-DCPH oxidation, experienced the highest peak currents by a significant margin (18.9 and 20.6 μA, respectively) at 0.57 V. The superior electrochemical performance of the CPA/GO-OXMWCNT and CPA/GO-OXSWCNT composites may be due to their high conductivity and specific surface area.

### 3.4. Cyclic voltammetric behavior

**Fig 10:**
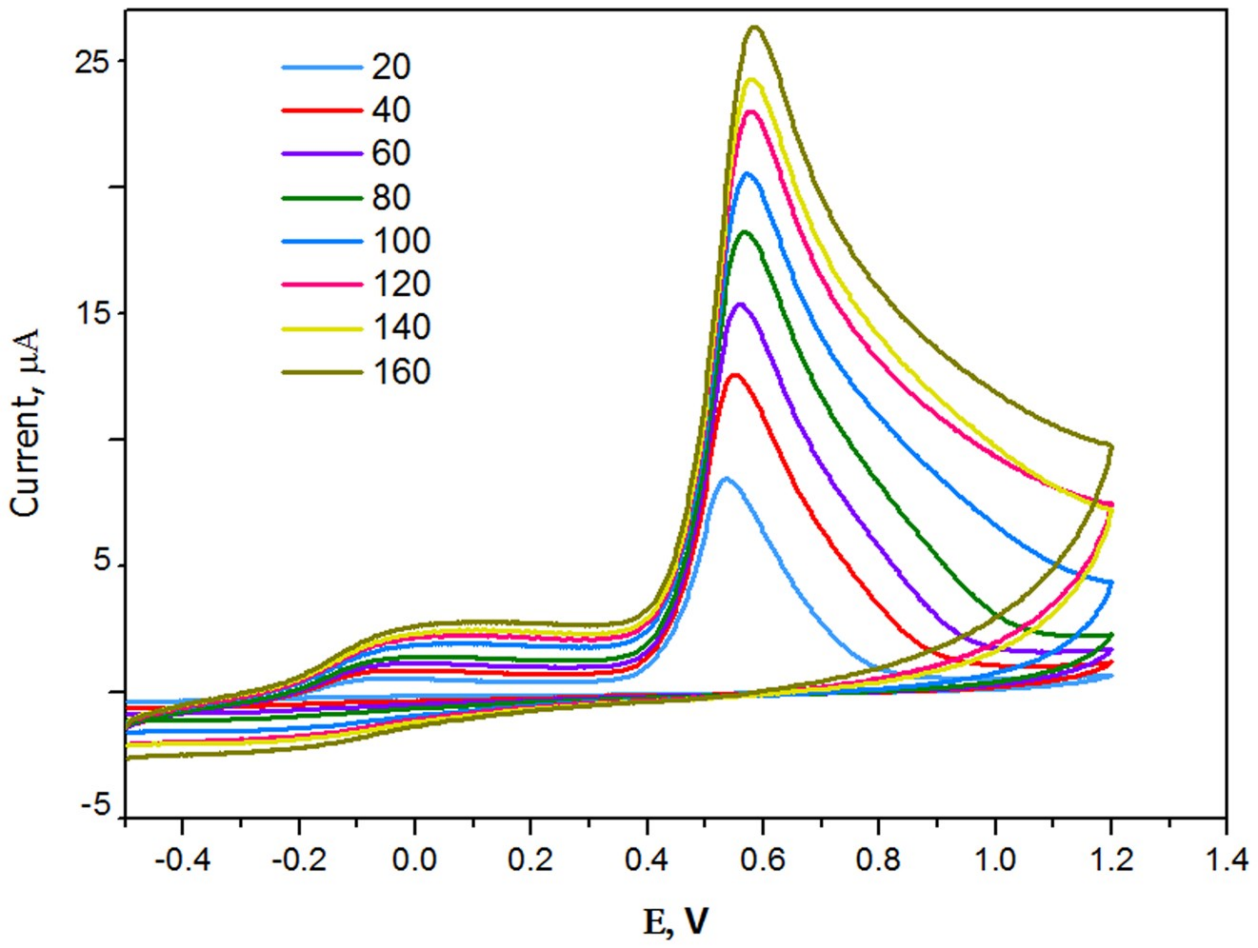
CV response of 1 ×10^−4^ mol L^-1^ 2,4-DCP at CPA/GO-OXSWCNTs/GCE with different scan rates from 20 to 160 in B–R buffer (pH 5.0).

CV is frequently utilized to obtain qualitative information on electrochemical reactions. Fig 10 shows the CV responses of the CPA/GO/OXSWCNTs/GCE when exposed to2,4-DCPH (1 × 10^−4^ mol L^-1^) in the B-R buffer(pH5) at various scan rates (20–160 mVs^-1^). The CV responses show a well-defined oxidation peak at the potential Epa = 0.54 V vs. Ag/AgCl reference electrode, which can be attributed to the oxidation of 2,4-DCPH to O-benzoquinone [51]. In the reverse scan, no cathodic peak was observed in the tested potential window, which confirms the irreversible nature of the electrochemical oxidation process. However, by increasing the scan rate, the anodic peak current steadily increased, which suggests a low chemical reaction and a limited mass transfer after the electrochemical process [52, 53]. The relationship between the anodic peak current (Ipa) and the square root of the sweep rate (ν) is shown in S2 Fig. Moreover, the linear regression equation can be stated as:

$$\text{Ipa}=1.9674\left({\text{v}}^{\text{0}\text{.5}}\right)+1.029;{\;\text{R}}^{2}=0.9949.$$ (1)

The current increased linearly with increasing scan rate, which shows that the oxidation process is controlled by diffusion [54, 55]. In addition, the effect of scan rate on the oxidation peak potential (Epa) was used to calculate the kinetic parameters, as shown in S3 Fig. The Epa increased with increasing scan rate, and there was a linear relationship between the Epa and ln *v*. The equation of linear regression is:

Epa=0.6364+0.0281lnv;R=0.9699, (2)

as reported by Laviron’s theory [52]. The Laviron equation can be used to describe the Epa of the irreversible electrode process:

$$\text{Epa}=\text{E}{}^{\circ}-\text{RT}/\left[\left(1-\alpha \right)\text{nf}\right]\;\text{ln}{\;\text{RTk}}_{\text{s}}/\left[\left(1-\alpha \right)\text{nf}\right]+\text{RT}/\left[\left(1-\alpha \right)\text{nf}\right]\;\text{ln}\;\text{v}.$$ (3)

**Fig 11:**
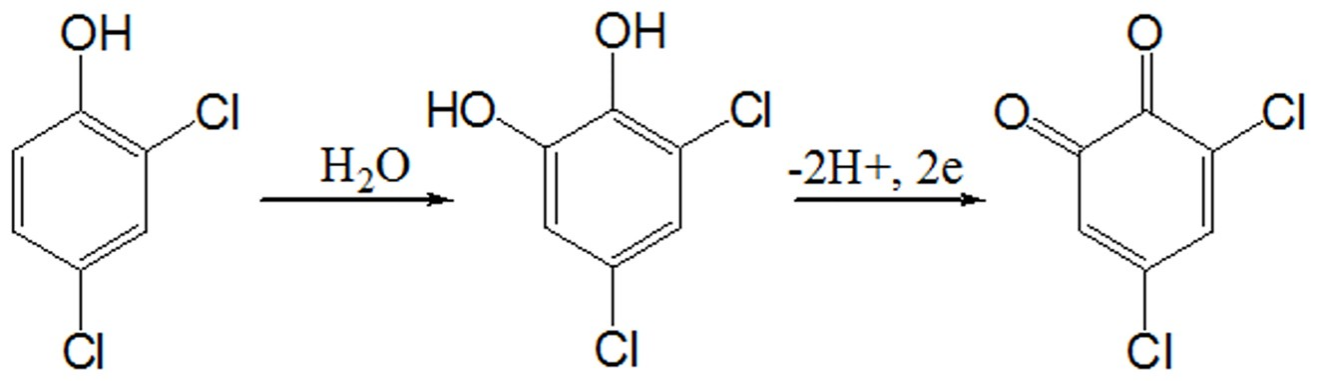
Oxidation mechanism of 2,4-DCP on modified electrode.

Based on this equation and given the slope of the linear plots of Epa of the anodic steps vs. lnν, the value of RT/(1-α) nF could be calculated. The value is presumed to be 0.5 for an absolutely irreversible electrode system [56]. Using T = 298 K, R = 8.314 J K^-1^ mol^-1^, F = 96480 C, and the value of the slope = 0.0281 (S3 Fig), the electron transmission number (n) for the oxidation of 2,4-DCPHwas calculated to be approximately 2. As such, the oxidation process of 2,4-DCPH on the CPA/GO/OXSWCNTs/GCE is a two-proton and two-electron process. Fig 11 shows the reaction process of 2,4-DCPH on this electrode.

### 3.5. Optimization of analytical parameters

**Fig 12:**
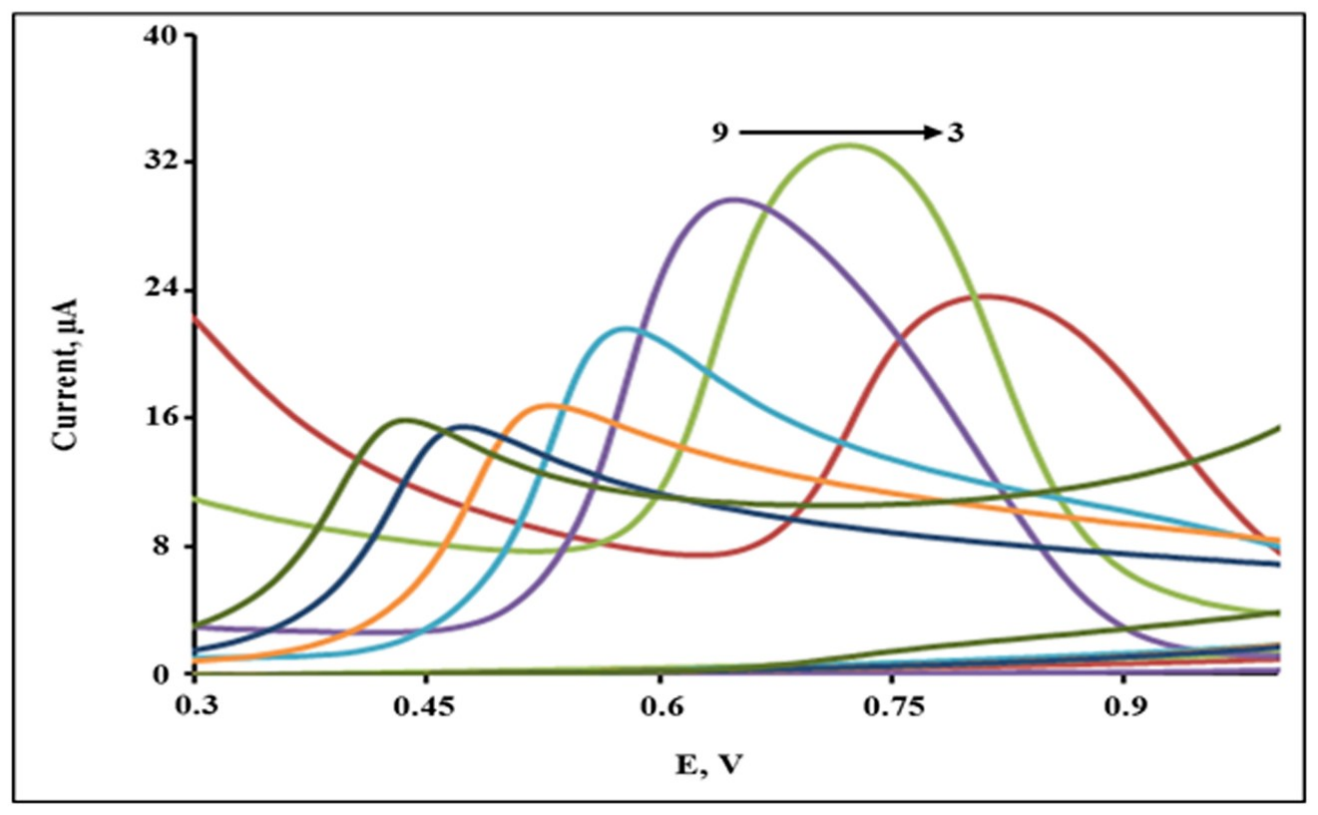
The CV responces of 1 × 10–4 mol L^−1^ 2,4-DCP on CPA/GO-OXSWCNTs/GCE in B–R buffer at different pH values (3.0, 4.0, 5.0, 6.0, 7.0, 8.0 and 9.0) with the scan rate of 50 mVs^-1^.

The impact of pH on the current of the oxidation peak of 2,4-DCPH (1 × 10^−4^ mol L^−1^)for the CPA/GO-OXSWCNTs/GCE was considered to be an important factor. Therefore, by utilizing CV, the influence of pH was investigated in the range of pH 3.0–9.0, as demonstrated in Fig 12. The maximum value of the oxidation peak current occurred at pH 4.0, and then decreased as pH increased further, as shown in S4 Fig. With the increase in pH from 3.0 to 9.0, it was observed that the oxidation peak potential shifted inversely, which exhibits the direct involvement of hydrogen ions in the oxidation process. The relationship between pH and Epa is presented in S5 Fig and the equation of linear regression is:

$$\text{Epa}=-0.0632\;\text{pH}+0.9721;{\;\text{R}}^{2}=0.9977.$$ (4)

The absolute values of the slope (V/pH)were close to the theoretical value of 0.059. This observation indicated that the number of electrons transferred was equal to the number of hydrogen ions that participated in the electrode reaction [51]. The optimum and well-defined peak current for 2,4-DCPH was obtained at pH 5, as shown in Fig 12. Therefore, the B–R buffer (pH 5.0) was utilized as the supporting electrolyte in all measurements.

### 3.6. Analytical properties

The detection reproducibility was investigated by determining the relative standard deviation (RSD) of eight duplicate measurements on a single electrode in 2,4-DCPH (1.0 × 10^−6^ mol L^-1^). It was determined that the RSD of peak currents for 2,4-DCPHwas 3.8%. Four GO/OXSWCNTs/GCEs were set up with the same configuration, and the 2,4-DCPH (1 μM) solution was detected by DPV under the optimized conditions. An RSD value of 6.1% was obtained for the experiment. The results showed that such factors did not impact the signals with RSDs < 7%. In addition, the stability of the CPA/GO-OXSWCNTs/GCEs was determined over a period of 25 days. After 25 days, the peak current of 2,4-DCPH (1 μM) retained 85.7% of its initial response. This finding signified that the modified electrode possessed good stability.

**Fig 13:**
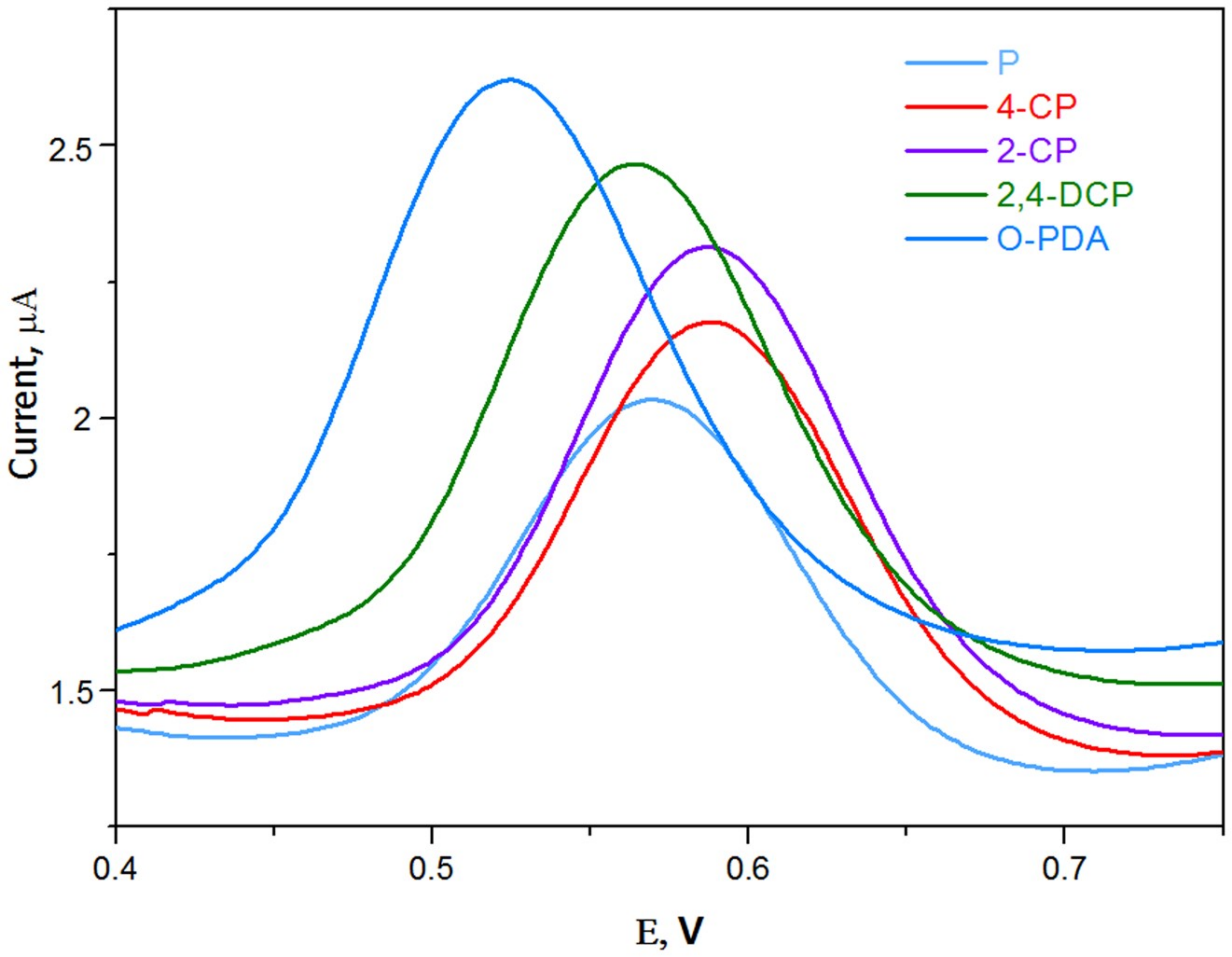
DPV responses of 2,4-DCP in presence of organic compounds.

To identify and investigate the possible analytical applications of the proposed sensor, the interference of some potential organic ions and inorganic compounds in real samples were examined by analyzing a standard solution of 2,4-DCPH (1.0 x 10^−6^ mol L^-1^) in the B-R buffer (pH 5.0), as presented in S6 Fig. Except for Mn^2+^ and Cu^2+^, the metal ions at large concentrations (1.0 × 10^−3^ mol L^-1^) had almost no noticeable impact on the peak current. Ethylenediaminetetraacetic acid (EDTA) was added to the detection system to eliminate Mn^2+^ interference with Cu^2+^. It was observed that the current of 2,4-DCPHremained constant when Mn^2+^ and Cu^2+^(1.0 × 10^−3^ mol L^-1^) were present in the B-R buffer after the addition of EDTA (3.0 × 10^−3^ mol L^-1^). The supporting electrolyte of the B-R buffer contained 3.0 × 10^−3^ mol L^-1^ of EDTA in these experiments. Considering that phenols with a similar structure may have similar electrochemical interferences with the determination of 2,4-DCPH, the electrochemical behaviors of common phenols (phenol, 2-CP, 4-CP, and o-PDA)were also assessed carefully under the same conditions. Fig 13 demonstrates that 2-CP and 4-CP exhibited similar electrochemical behaviors to that of 2,4-DCPH. However, their oxidation potentials (0.59 V) were different from those of 2,4-DCPH, and the o-PDA oxidation potential (0.53 V) was also significantly different from that of 2,4-DCPH. Although the electrochemical signals of 2,4-DCPH were much larger than those of phenol, their oxidation potential values were very similar to one another. Therefore, the CPA/GO-OXSWCNT/GCE sensor may have the potential to selectively determine chlorophenols.

**Fig 14:**
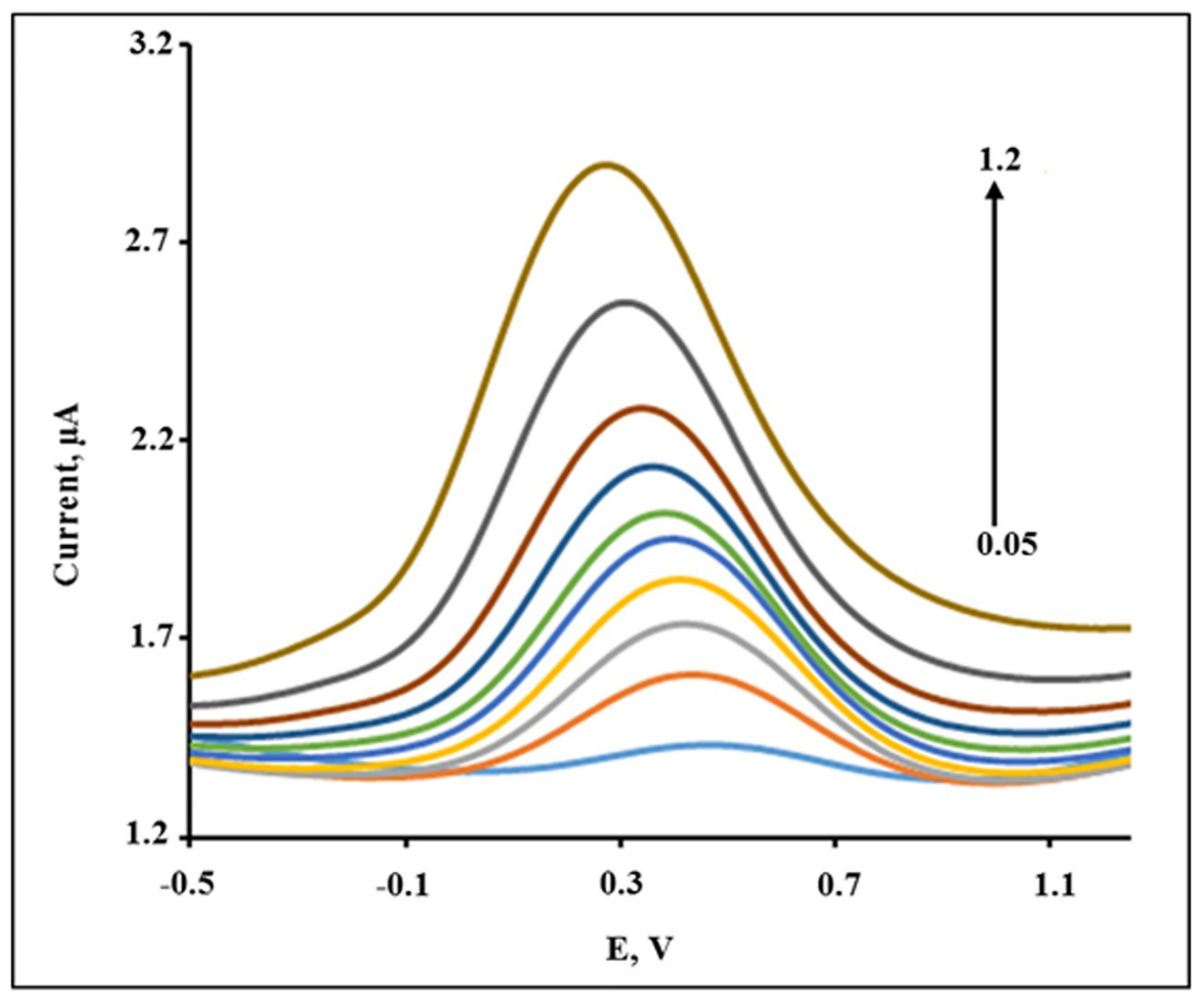
DPV of different concentration of 2,4-DCP: 0.05, 0.1, 0.2, 0.3, 0.4, 0.5, 0.6, 0.7, 0.9 and 1.2 ×10^−6^ mol L^-1^ on the CPA/GO-OXSWCNTs/GCE in B-R buffer (pH 5.0).

As an electrochemical method, DPV has a much higher current sensitivity and better resolution compared to CV [57]. Therefore, DPV was used to determine the electrochemical response of 2,4-DCPHat different concentrations of CPA/GO-OXSWCNTs/GCE under the optimized conditions. Fig 14 shows that the oxidation peak currents of 2,4-DCPH gradually increase as the concentration increases from 0.05 to 1.2 × 10^−6^ mol L^-1^. The relationship between the peak current (I) and concentration (c) is shown in S7 Fig and the equation of linear regression is:

$$\text{I}=1.1873\;\text{c}\;1.4403;{\;\text{R}}^{2}=0.9927.$$ (5)

The detection limit (LOD) can be expressed as:

LOD=3Sa/b, (6)

where Sa is the standard deviation of the response and b is the calibration curve slope. The LOD for 2,4-DCPH was determined to be approximately 4.2 × 10^−6^ mol L^-1^. Compared with other electrochemical sensors [58–65], the fabricated CPA/GO-OXSWCNTs/GCE exhibited a relatively high sensitivity and wide linear range (Table 3). For practical applications, the CPA/GO-OXSWCNTs/GCE sensor was employed to detect 2,4-DCPH in fish farm water samples by the standard addition method. The water was filtered and adjusted to pH 5.0 with the B-R buffer. The recoveries and RSDs were measured and are shown in Table 4. The recoveries and RSD for 2,4-DCPH were calculated as 95.3–104% and 2.41–3.5%, respectively. This demonstrated that the electrode can be applied to the analysis of real samples.

**Table 3:** Performance comparison of the fabricated electrode for 2,4-DCP detection with the other reported electrodes.

Electrode	Technique	Linear Range (μmol L^-1^)	LOD(mol L^-1^)	Ref
MWCNT/Nafion/GCE	DPV	1–150	1.0 × 10^−8^	[13]
CS/CDs–CTAB/GCE	DPV	0.04–8	10 × 10^−9^	[25]
Cu3(BTC)2–CPE	DPV	0.04–1.0	9.0× 10^−9^	[63]
IL-CNNS/GCE	Amperometry	0.02–160	6.2 × 10^−9^	[64]
β-CDBIMOTs/CPE	Amperometry	4–100	1.2 × 10^−6^	[16]
HRP/MWNTs/GCE	Amperometry	1.0–100	3.8 × 10^−7^	[17]
MIP/GCE	DPV	5–100	1.6 × 10^−6^	[65]
SWCNT/PEDOT/GCE	Amperometry	0.4–120	2.28 × 10^−7^	[19]
GO/OXSWCNTs/GCE	DPV	0.05–1.2	4.2 × 10^−9^	Present work

MWCNT/Nafion/GCE: multi-walled carbon nanotubules Nafion glassy carbon electrode, CDs–CTAB–CS/GCE: carbon dots, hexadecyltrimethyl ammonium bromide and chitosan/glassy carbon electrode, Cu3(BTC)2–CPE: 1,3,5-benzenetricarboxylic acid copper-modified carbon paste electrode, IL-CNNS/GCE: ionic liquid nanostructure via a nucleophilic substitution between the tertiary amino groups in tri-s-triazine subunits and n-butyl bromide/glassy carbon electrode, β-CDBIMOTs/CPE: cyclodextrin-functionalized ionic liquid modified carbon paste electrode, HRP/MWNTs/GCE: Horseradish peroxidase—multiwalled carbon nanotubues modified glassy carbon electrode, MIP/GCE: molecularly imprinted polymer–glassy carbon electrode, SWCNT/PEDOT/GCE: Single-walledcarbonnanotubespoly(3,4-ethylenedioxyth-iophene) modified glassy carbonelectrode.

**Table 4:** Results of 2,4-DCP determination in fish farm water analysis.

Sample	2,4-DCP added (μ mol L^-1^)	2,4-DCP Found (μ mol L^-1)^^a^	Recovery (%)^b^
Fish farm water	0.05	0.052	104 ± 3.17
	0.1	0.103	103 ± 3.44
	0.5	0.489	97.8 ± 3.50
	1.0	0.960	96.0 ± 2.41
	2.0	1.905	95.3 ± 2.65

^a^ Average of five measurements

^b^ Average of recovery ± standard deviation

## 4. Conclusion

In this study, CPA/OXCNM NCs were synthesized by the oxidative chemical polymerization method and used to fabricate an electrochemical sensor to detect 2,4-DCPHin a fish farm water sample. The introduction of both types of OXCNTs and GO with high conductivity considerably increased the electrochemical performance of the CPA electrodes. Compared with CPA/GO-OXMWCNT NCs, CPA/GO-OXSWCNTs showed considerably improved electrochemical performance. In the as-prepared CPA/GO-OXSWCNT NCs and CPA/GO-OXMWCNT NCs, the mixture of OXCNMs created highly interconnected networks which offered fast electron and charge transport paths to achieve high electrical conductivity. In addition, the GO-supported CPA-coated OXCNT ternary hybrid microstructure exhibited a high surface area and increased electrical conductivity. In comparison to bare GCE and other NCs, the oxidation peak current of 2,4-DCPH was significantly increased for the CPA/GO-OXSWCNTs/GCE sensor. The electrochemical behavior of 2,4-DCPH on the modified electrode is a diffusion-controlled process that involves two electrons with two proton transfers. The prepared CPA/GO-OXSWCNTs/GCE sensor exhibited strong catalytic activity for 2,4-DCPH oxidation and exhibited a favorable quantitatively reproducible analytical performance. This novel method has certain clear advantages such as simplicity, low-cost, rapid response, and high sensitivity.

## Supporting information

S1 Fig DTG curves of CPA and CPA/OXCNMsNCs. (DOCX)

S2 Fig Plot of Ipa vs. square root of the scan rate (ν ^1/2^) of 1 × 10^−4^ mol L^−1^ 2,4-DCP at pH = 5. (DOCX)

S3 Fig Plot of Ep,a vs. lnν for anodic peak of 2,4-DCP at pH 5. (DOCX)

S4 Fig The peak current (Ipa) of 1 × 10^−4^ mol L^−1^ 2,4-DCP in in B–R buffer at different pH values at the CPA/GO-OXSWCNTs/GCE. (DOCX)

S5 Fig The oxidation peak potential with respect to pH; scan rate 50 mVs^-1^. (DOCX)

S6 Fig Column chart of the oxidation peak current of 1 ×10–6 mol L^-1^ 2,4-DCP in B-R buffer solution (pH of 5.0), containing 1.0 ×10^−3^ mol L^-1^ metal ions. (DOCX)

S7 Fig The calibration curve of the DPV responses of 2,4-DCP concentration. (DOCX)

S1 Graphical Abstract (TIF)

## Abbreviations

2,4-DCP: 2,4-Dichlorophenol
2-CP: 2-Chlorophenol
4-CP: 4-Chlorophenol
Ani: Aniline
APS: Ammonium persulfate
CDT_*final*_: The final composite degradation temperature
CP: Conducting polymer
CPA: Crosslinked polyaniline
CPHs: Chlorophenols
CV: Cyclic voltammetry
DMF: Dimethylformamide
DPV: Differential pulse voltammetry
DTG: Derivative thermogravimetry
FTIR: Fourier transform infrared
GCE: Glassy carbon electrode
GO: Graphene oxide
H_2_SO_4_: Sulphuric acid
HCl: Hydrochloric acid
HNO_3_: Nitric acid
HR-TEM: High-resolution transmission electron microscope
NCs: Nanocomposites
NPs: Nanoparticles
o-PDA: o-Phenylenediamine
OXCNMs: Oxidized carbon nanomaterials
OXMWCNTs: Oxidized multi-walled carbon nanotubes
OXSWCNTs: Oxidized single-walled carbon nanotubes
PANI: Polyaniline
PDT_*max*_: The maximum polymer degradation temperature
PPDA: p-phenylenediamine
RSDs: Relative standard deviations
SEM: Scanning electron microscopy
T_10_: Temperatures corresponding to the decomposition of 10% weight loss
T_25_: Temperatures corresponding to the decomposition of 25% weight loss
T_50_: Temperatures corresponding to the decomposition of 50% weight loss
TGA: Thermogravimetric analysis
TPA: triphenylamine
