# Supplementary figures and images for: Rapid and sensitive electrochemical sensor of cross-linked polyaniline/oxidized carbon nanomaterials core-shell nanocomposites for determination of 2,4-dichlorophenol
Source: PLoS One. 2020 Jun 25;15(6):e0234815. doi: 10.1371/journal.pone.0234815 (PMC7316237; doi:10.1371/journal.pone.0234815)

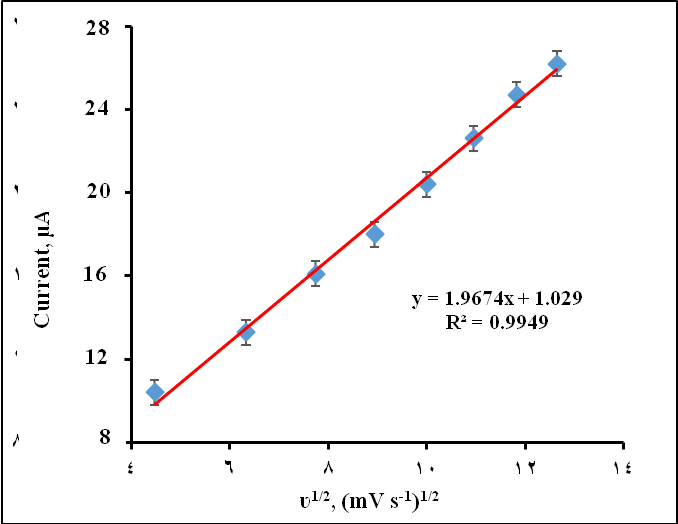


**S2 Fig.**

Supplement: S2 Fig — (DOCX) [file pone.0234815.s002.docx]

**
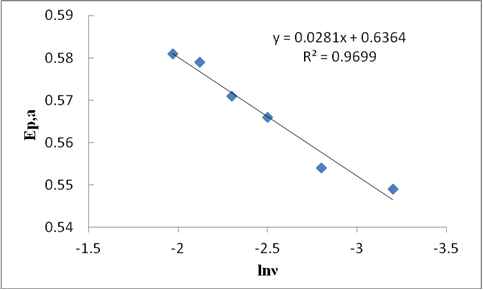
**

**S3 Fig.**

Supplement: S3 Fig — (DOCX) [file pone.0234815.s003.docx]

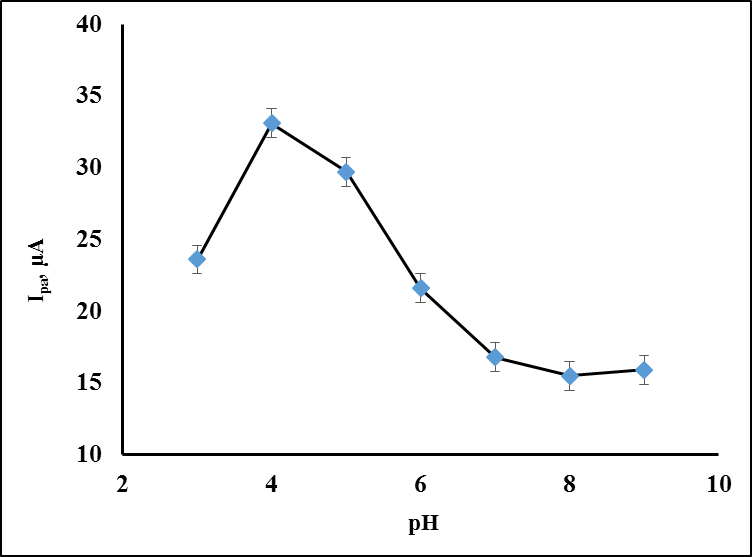


**S4 Fig.**

Supplement: S4 Fig — (DOCX) [file pone.0234815.s004.docx]

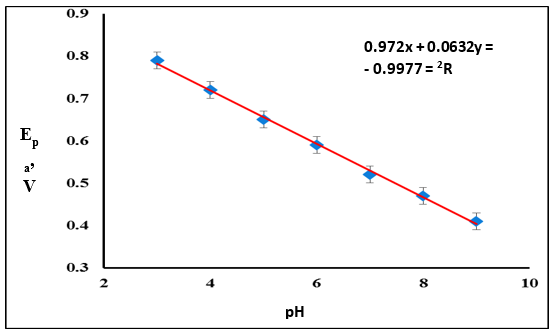


**S5 Fig.**

Supplement: S5 Fig — (DOCX) [file pone.0234815.s005.docx]

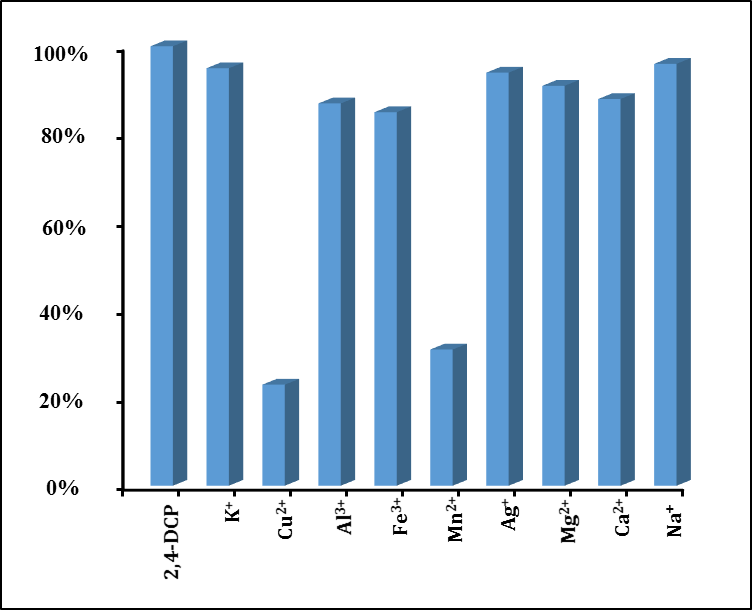


**S6 Fig.**

Supplement: S6 Fig — (DOCX) [file pone.0234815.s006.docx]

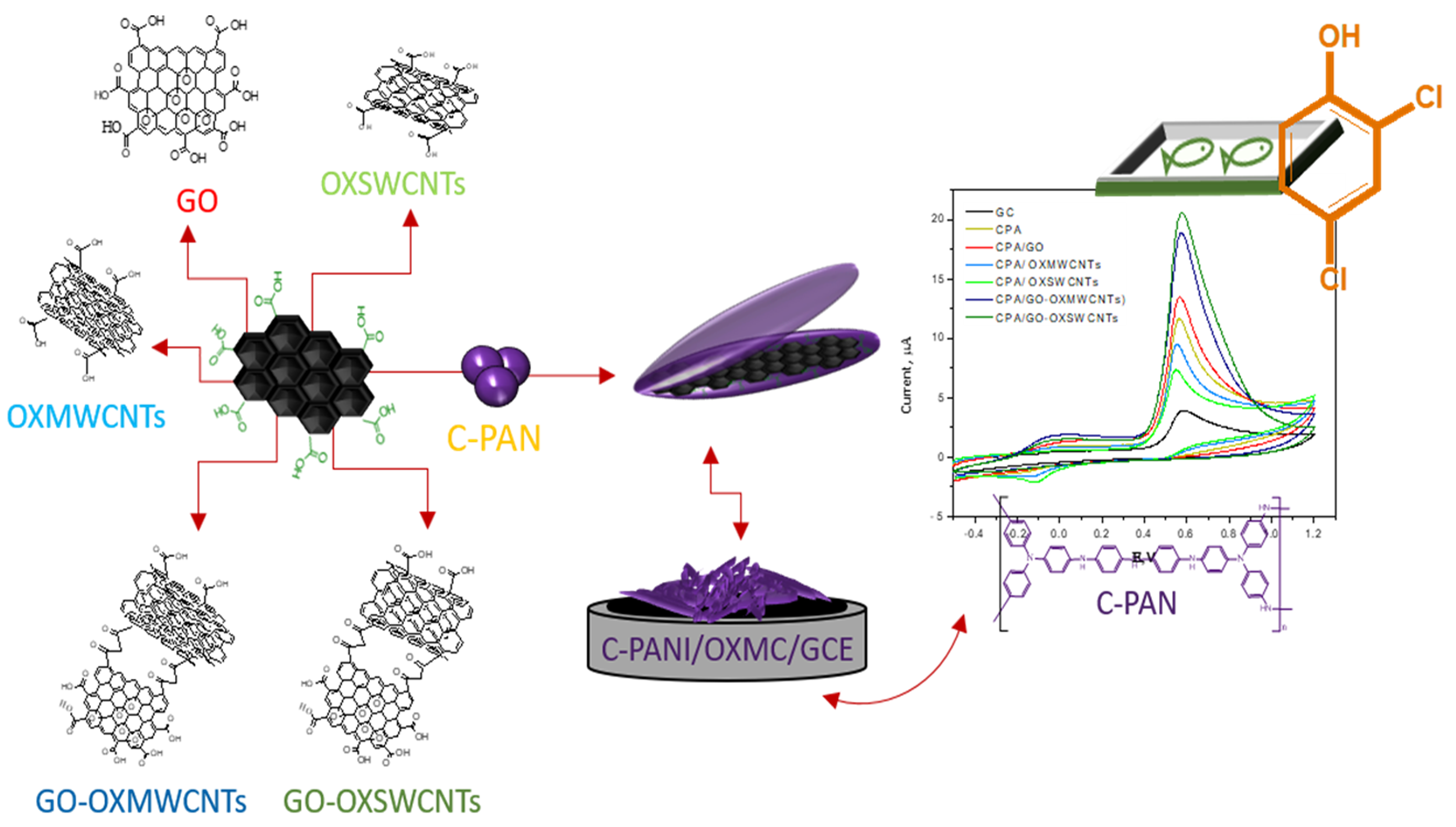

Supplement: S1 Graphical Abstract — (TIF) [file pone.0234815.s008.tif]
