# Supplementary material for: Rapid and sensitive electrochemical sensor of cross-linked polyaniline/oxidized carbon nanomaterials core-shell nanocomposites for determination of 2,4-dichlorophenol
Source: PLoS One. 2020 Jun 25;15(6):e0234815. doi: 10.1371/journal.pone.0234815 (PMC7316237; doi:10.1371/journal.pone.0234815)
Supplement: S7 Fig — (DOCX) [file pone.0234815.s007.docx]

**Current, µA**

**S7 Fig.**
